# Subtraction Ictal SPECT coregistered to MRI (SISCOM) as a guide in localizing childhood epilepsy

**DOI:** 10.1002/epi4.12373

**Published:** 2019-12-26

**Authors:** Thomas Foiadelli, Lieven Lagae, Karolien Goffin, Tom Theys, Mara De Amici, Lucia Sacchi, Johannes Van Loon, Salvatore Savasta, Katrien Jansen

**Affiliations:** ^1^ Pediatric Clinic Fondazione IRCCS Policlinico San Matteo University of Pavia Pavia Italy; ^2^ Department of Development and Regeneration University Hospitals Leuven Leuven Belgium; ^3^ Nuclear Medicine and Molecular Imaging University Hospitals Leuven KU Leuven Leuven Belgium; ^4^ Neurosurgery Department University Hospitals Leuven Leuven Belgium; ^5^ Laboratory of Immuno‐Allergology Fondazione IRCCS Policlinico San Matteo Pavia Italy; ^6^ Department of Electrical, Computer, and Biomedical Engineering University of Pavia Pavia Italy

**Keywords:** epilepsy, epilepsy surgery, focal cortical dysplasia, focal seizures, SISCOM, SPECT

## Abstract

**Objective:**

To assess feasibility and efficacy of subtraction ictal SPECT coregistered to MRI (SISCOM) for epilepsy localization in children who are candidates for resective surgery.

**Methods:**

We retrospectively reviewed all patients ≤16 years with drug‐resistant epilepsy screened for epilepsy surgery in the University Hospital of Leuven from January 2009 to January 2018. Fifty‐eight hospitalizations for ictal SPECT and 51 SISCOM analyses in 44 patients were included. Mean age was 9.1 years. Hospitalizations for SISCOM were analyzed in terms of multiple variables affecting feasibility and efficacy. The localization of SISCOM was compared with the localization of the presumed epileptogenic zone (PEZ) as determined by video‐EEG.

**Results:**

SISCOM was feasible in terms of chronic medication management, rescue antiepileptic therapy during hospitalization, and operative timings. Radiotracer injection occurred within 30 seconds from seizure onset in 91.4% of the patients. ictal SPECT imaging was performed within two hours from injection in 100% of the patients (mean: 40 minutes). SISCOM was able to localize the PEZ in 51.0% (26/51) and to additionally lateralize the PEZ in 17.6% (9/51), achieving better localizations than ictal SPECT, FDG‐PET, and MRI (*P* < .01). SISCOM was useful to localize the PEZ in 25% of patients with poorly localizing video‐EEG and in 27.8% of MRI‐negative cases. The occurrence of habitual seizures during injection for ictal SPECT and the temporal localization of the PEZ both correlated with a better SISCOM localization (*P* < .05). 36.4% (16/44) patients were finally selected for resective surgery, with a 87.5% seizure‐free rate at 12 months. A localizing SISCOM was associated with seizure freedom in 66.7% and with a Engel I‐II in 75.0% of our patients.

**Significance:**

SISCOM is a reliable tool to localize the epileptogenic zone in clinical practice and is both feasible and useful in children, adding precious presurgical information especially in patients with noninformative MRI or a poorly localizing video‐EEG.

**Key Points**

- SISCOM is feasible, even in young children, and provides good localization of the presumed epileptogenic zone (PEZ) for presurgical evaluation
- SISCOM achieved significantly better localization of the PEZ than MRI, PET, and ictal SPECT
- SISCOM was particularly useful in cases with noninformative EEG or MRI
- A localizing SISCOM correlated with a better postsurgical outcome in terms of seizure freedom at 12 months

## 1 INTRODUCTION

Despite the exponential discovery of new antiepileptic drugs in the last decades, approximately one‐third of all children with epilepsy remains drug‐resistant. Remarkably, the likelihood of achieving seizure control decreases with each unsuccessful AED regimen and becomes very narrow (<1%) after the third AED.^1^ For this reason, children with drug‐resistant epilepsy must undergo specific investigations to assess eligibility for alternative therapeutic options, notably epilepsy surgery.^2^, ^3^, ^4^ Recent randomized, blinded controlled trials have provided level I evidence for large benefits from early epilepsy surgery in adolescents and children with drug‐resistant focal epilepsy.^2^, ^3^, ^5^

In the presurgical evaluation of these patients, the epileptogenic zone (EZ) must be precisely delineated.^6^ 3‐Tesla‐MRI has become an essential tool to identify epileptogenic lesions. Unfortunately, in a considerable number of patients, no clear lesion can be identified.^7^ This is particularly true in children, in whom extratemporal lobe epilepsy is more frequent, small epileptogenic lesions (ie, focal cortical dysplasia) can be very hard to identify even by expert radiologists, and the presence of other nonepileptogenic lesions (eg, tubers or large degenerative areas) can lead to misinterpretations.^8^, ^9^ Finally, the EZ does not always correspond to (or is not limited to) the epileptogenic lesion.^10^ At present, the gold standard for the identification of the EZ is invasive EEG.^11^ However, placement of intracranial and in particular subdural electrodes has a considerable burden of perioperative complications and several limitations in children.^12^ For these reasons, validation of noninvasive imaging techniques applied to the pediatric population for the identification of the EZ is essential.^13^ Subtraction ictal SPECT coregistered to MRI (SISCOM) is a high‐resolution imaging tool that provides high sensitivity and specificity localization of the seizure focus^14^ with a remarkable predictive value of good surgical outcome.^15^ SISCOM has been used with variable positive outcomes in adults,^7^, ^16^, ^17^, ^18^ but large studies applied to the pediatric population are lacking.^19^ The scope of the present study is to assess the feasibility of SISCOM in a pediatric clinical setting and to determine whether it is useful to localize the presumed epileptogenic zone (PEZ) in children who are candidate for resective surgery.

## 2 METHODS

This is a retrospective single‐center cohort study. All patients entering the Epilepsy Surgery protocol in the Pediatric Neurology Unit of the University Hospital of Leuven from January 2009 until January 2018 were included in the study. Inclusion criteria were the following: (1) drug‐resistant focal epilepsy or generalized or multifocal epilepsy with a predominant unifocal onset; (2) complete presurgical evaluation including: (a) clinical and neurocognitive assessment, (b) ≥24‐hour video‐EEG monitoring (BrainRT—OSG), (c) 3‐Tesla brain‐MRI with an 8‐channel phased‐array head coil (Philips), and (d) SISCOM. Exclusion criteria were as follows: (1) age ≥17 years old and (2) incomplete data in ≥1 presurgical examinations.

### 2.1 SISCOM protocol

All patients entering the epilepsy surgery protocol were scheduled for a 5‐day hospitalization in the EEG monitoring unit for continuous video‐EEG recording, with the aim to detect a seizure suitable for ictal SPECT. ^99m^Tc‐ethyl cysteinate dimer (^99m^Tc‐ECD) was used as intravenous tracer, upon seizure occurrence. Deep sedation was provided for SPECT in children <8 years old and/or unable to cooperate. Hospitalization could be extended for an additional ictal SPECT (at least 48 hours apart, to allow a complete washout of the tracer), whenever necessary. In all cases, potential harms and benefits of the exposure to radiations and eventually sedation for diagnostic purposes were discussed with the parents, and written informed consent was collected before the procedure.

**Figure 1:**
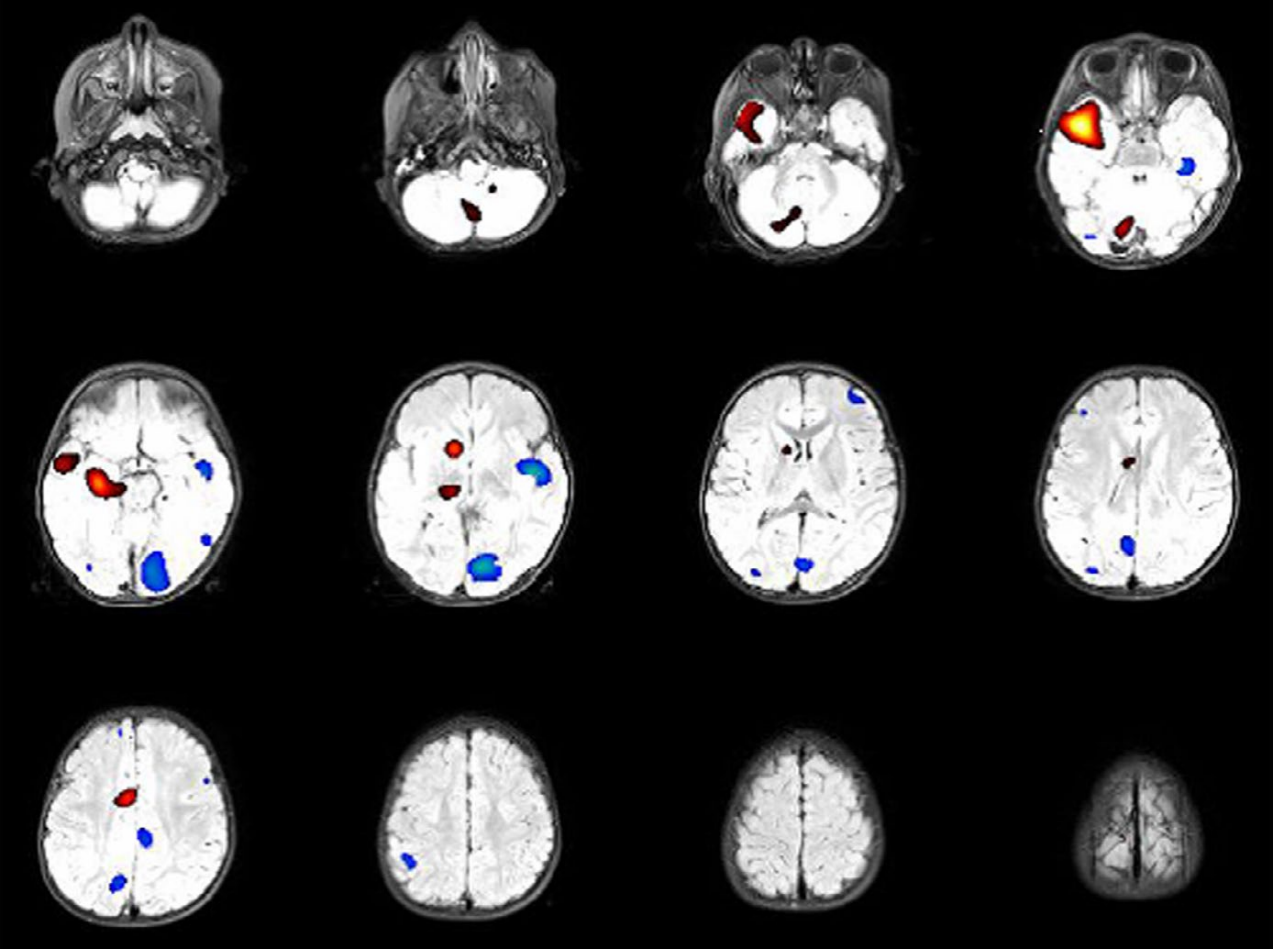
Subtraction ictal SPECT coregistered to MRI (SISCOM) imaging. Relative ictal hyperperfusion (+2 to +4 SD) is identified in red‐yellow, while the relative ictal hypoperfusion zones are colored in blue (−2 to −4 SD). Notice the localized zone of relative hyperperfusion in the right anteromesial temporal lobe (yellow), with propagation to the superior lateral temporal neocortex (red), while the contralateral anteromesial temporal lobe shows a light hypoperfusion (light‐blue). The patient reached complete seizure freedom after resective surgery, which confirmed the presence of a focal cortical dysplasia type IIA in the right anterior temporal lobe (patient #39)

SPECT images are acquired using IRIX Prism (Philips Medical Systems) until 2010 and using Discovery NM‐CT 670 (GE Healthcare) afterward. Ictal and interictal SPECT studies were finally coregistered using the registration module of Statistical Parametric Mapping (SPM version 8; Wellcome Trust Centre for Neuroimaging), implemented in Matlab (R2012; The MathWorks Inc). A *z*‐score threshold of 2.0 was considered to have the optimal specificity and sensitivity for ictal onset zone localization.^20^ SISCOM image was created coregistering the average of both SPECT images to the preoperative MRI, using MRICRO software (Georgia Institute of Technology, Atlanta, USA) for detailed anatomical localization (Figure 1). Comprehensive operative protocols and technical details for ictal SPECT, including provocative techniques and sedation, and SISCOM are available as supplementary material (Data S1 SISCOM protocol).

In all patients, resective surgery feasibility, strategy, and eventually extent of resection were determined by multidisciplinary consensus with integration of all clinical, paraclinical, and technical investigations. In some cases, ^18^FDG‐PET was performed to support the hypothesis. Informed written consent was obtained from all patients and/or their parents or legal tutors, and all procedures were approved by the Hospital Ethics Committee.

### 2.2 Data collection and analysis

Data were derived from electronic clinical records. Timings of clinical acts during hospitalization and SPECT were determined on retrospective video and EEG review. Only seizures in which injection for ictal SPECT was performed were considered for data analysis.

Seizure onset and ending was defined, respectively, as the earliest and the last ictal EEG or clinical evidence of seizure activity. Injection during a generalization was a priori defined as a primary or secondary generalization on EEG starting anytime before the end of injection. Seizures were defined as “nonhabitual” if ictal clinical and EEG data were discordant from previous clinical and video‐EEG recordings.

All data were reviewed by two experienced pediatric epileptologists (LL and KJ). The PEZ was determined by consensus, blinded from personal and imaging data, taking into account video‐EEG monitoring data, as well as ictal semiology and clinical features, and assigned to one of eight cerebral lobes (left or right frontal, temporal, parietal, and occipital lobe) whenever possible.

Feasibility outcomes were defined in terms of the following: (a) number of hospitalizations, (b) successful rate of ictal SPECT hospitalizations, (c) median hospitalization stay, (d) adverse events and need for rescue medication, (e) timings for ictal and interictal SPECT performance, including radiotracer injection time and delay from injection to SPECT acquisition, and (f) number of SISCOM analyses with a positive outcome (localizing studies). Imaging outcome was defined as “positive” or “negative” according to its capability to localize the epileptic focus to one single lobe (or part of a lobe). SISCOM outcome was compared to the outcomes of other imaging techniques commonly used in the presurgical evaluation of adults and children with epilepsy (ie, 3T‐MRI, ictal SPECT, FDG‐PET).

The following concepts were used to classify neuroimaging results. Studies were defined as *localizing* when abnormal findings were concordant with the lobar location of the PEZ and *highly localizing* when they provided additional information on the sublobar localization of the PEZ (eg, mesial or lateral temporal lobe, precentral gyrus). When the abnormal finding was identified in the same hemisphere but not in the same lobe with regard to the PEZ, the study was considered *lateralizing*. All other cases were defined as *noninformative*. These included the following: normal studies (the absence of abnormal findings) and nonconcordant studies (abnormal findings discordant with the brain location of the PEZ). When the PEZ could not be determined in a single lobe based on EEG, the study was defined as *localizing* if abnormal findings were identified in one definite lobe, *lateralizing* if two contiguous ipsilateral lobes were involved, or otherwise *nonlateralizing*. However, if the final PEZ after multidisciplinary consensus was considered discordant with this localization, the study was defined as *falsely localizing*/*falsely lateralizing.*

Data analysis was performed using MatlabR2017a (The Mathworks, Inc). Quantitative variables were summarized as mean and standard deviation (SD), and categorical variables were summarized as a number (%). Distributions of categorical variables between subgroups were compared using the chi‐square test, whereas the Wilcoxon test was used to compare quantitative variables. Statistical significance was considered for all tests at *P* < .05.

## 3 RESULTS

### 3.1 Demographic and clinical data

**Figure 2:**
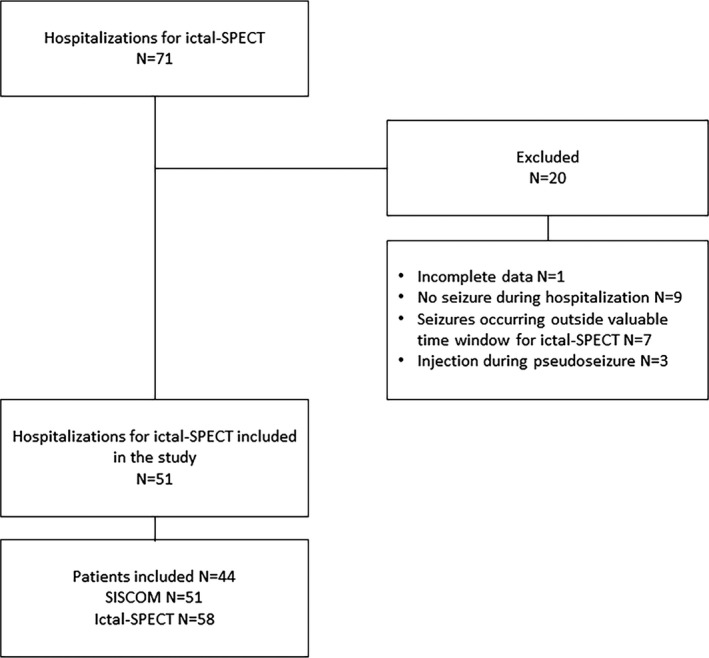
Study flow chart

Data from 71 hospitalizations for ictal SPECT in patients <17 years old were collected from January 2009 to January 2018. One case was excluded for incomplete EEG/clinical data and 19 for ictal SPECT not being performed (Figure 2). Fifty‐one hospitalizations (71.8%) were thus considered for data analysis. These included 58 ictal SPECT in 44 pediatric patients: 24 males (54.5%) and 20 females (45.5%). Mean age was 9.1 years (1‐16 years), with 11 patients (25%) younger than 6 years. Mean age at epilepsy onset was 4.8 years (1 month‐13 years), and the average time from epilepsy onset to ictal SPECT was 54 months (2‐195 months).

Based on clinical and EEG data, we could define the PEZ in 33/44 (75.0%) patients. Twelve patients had temporal lobe epilepsy (27.3%), five patients had frontotemporal localization (11.4%), and 16/44 (36.4%) had extratemporal localization (frontal, n = 13; frontoparietal, n = 1; parietal, n = 2).

Demographic and clinical characteristics are reported in Table 1. Analytical data are available in Table S1.

**Table 1:** Clinical and demographic characteristics

Patient n./Sex	Age	Etiology	Seizures classification^b^	PEZ^a^	MRI^a^	SISCOM^a^	SISCOM outcome	Surgery	Follow‐up (mo)	Final outcome (Engel class)
1/F	14	Structural: MTS	Focal autonomic impaired awareness	T/L	T/L	T/L	Highly localizing	Yes	119 (101 post‐S)	Class I
2/M	15	Genetic: Fragile X Structural: MTS	Focal tonic	T/L	T/L	T/L	Highly localizing	Yes	117 (43 post‐S)	Class I
3/F	10	Structural: FCD	Focal tonic, impaired awareness	T/R	T/R	T/R	Localizing	No	108	Class I
4/F	9	Structural: FCD	Focal clonic	T/R	T/R	T/R	Highly localizing	Yes	106 (96 post‐S)	Class I
5/F	3	Structural: FCD	Focal tonic	F/R	F/R	F/R	Localizing	Yes	105	Class III
6/M	12	Structural: MTS	Auditory aura + focal impaired awareness	T/R	T/R (MTS) + P/R	T/R	Highly localizing	Yes	105 (100 post‐S)	Class I
7/M	11	Structural: MCA stroke + MTS	Focal impaired awareness	None	F‐P‐T/L	T/R	Localizing	No	97	Class IV
8/M	14	Structural: FCD	Focal clonic aware, to GTC	F/R	F/R	F/R	Highly localizing	No	96	Class IV
9/M	4	Structural: FCD	Focal tonic	F/R	F/R	F/R	Highly localizing	Yes	95 (90 post‐S)	Class I
10/M	7	Immune: HHE	Three types: 1) focal tonic, 2) motor arrest, 3) myoclonic	Mult	F‐T‐P/L	None	Noninformative	No	86	Class III
11/M	15	Structural: FCD	Focal tonic to GTC	T/L	T/L	T/L	Localizing	Yes	79 (74 post‐S)	Class III
12/F	1	Structural: FCD	Epileptic spasms + focal non motor impaired awareness	F/R	F/R	None	Noninformative	Yes	78 (74 post‐S)	Class I
13/F	5	Genetic: TSC Structural: FCD	Multifocal: focal tonic impaired awareness to GTC, + myoclonic	Mult	Bilat T	T‐P/R	Lateralizing	No	74	Class II
14/F	13	Unknown	Focal tonic	Bilat F	Normal	T/R	Noninformative	No	73	Class III
15/M	7	Immune: AE	Focal clonic	F/L	T/L	F/L	Normal	No	72	Class IV
16/F	9	Unknown	Focal motor to GTC + focal cognitive with automatism.	Bilat F‐T	Normal	Bilat nucleus caudatus	Noninformative	No	71	Class I
17/F	16	Structural: FCD	Focal with autonomic aura, impaired awareness	T/R	T/R	T/R	Localizing	Yes	70 (64 post‐S)	Class I
18/M	4	Unknown	Nocturnal hypermotor with automatisms	F/R	Normal	None	Noninformative	No	68	Class I
19/M	14	Structural: FCD	Focal impaired awareness to GTC	F/L	F/L	F/L	Localizing	No	63	Class I
20/M	10	Unknown	Focal clonic aware to GTC	Bilat F	Normal	F‐P/R	Lateralizing	No	60	Class III
21/F	8	Unknown	Focal cognitive	Mult	F/R	None	Noninformative	No	56	Class III
22/M	11	Structural: FCD	Focal cognitive + oral automatisms	Bilat T	P/R	P/R	Localizing	No	55	Class III
23/M	16	Structural: FCD	Focal motor impaired awareness to GTC	F‐T/R	F/R	F‐O/R	Lateralizing	No		
24/F	8	Immune: FIRES	Focal clonic impaired awareness	F/L	Normal	F‐T/L	Lateralizing	No	54	Class II
25/F	6	Structural: FCD	Focal autonomic impaired awareness	T/R	T/R	T/R	Highly localizing	Yes	51 (41 post‐S)	Class I
26/M	12	Structural: Ganglioglioma	Focal cognitive + clonic	T/R	T/R	T/R	Localizing	Yes	50 (46 post‐S)	Class I
27/F	7	Genetic: PAI syndrome	Focal sensory + tonic	P/L	Normal	F‐P/L	Lateralizing	No	50	Class II
28/M	8	Unknown	Focal clonic aware	F‐P/R	Normal	High P/R	Highly localizing	No	44	Class I
29/M	12	Unknown	Focal tonic to GTC	F/L	Normal	Mid‐F on the midline	Highly localizing	No	43	Class I
30/F	7	Unknown	Focal clonic	F/R	Normal	F/R	Highly localizing	No	39	Class III
31/M	13	Unknown	Focal non motor impaired awareness	F‐T/R	Normal	F‐P/L	Noninformative	No	38	Class II
32/M	10	Structural: Oligodendroglioma	Focal sensory aware	T/R	T/R	Bilat T	Noninformative	Yes	34 (33 post‐S)	Class I
33/M	10	Structural: MTS	Focal motor impaired awareness	T/R	T/R	T/R	Highly localizing	Yes	32 (25 post‐S)	Class I
34/M	15	Structural: FCD	Nocturnal focal hyperkinetic	F/R	F/R	F/R	Highly localizing	Yes	31 (12 post‐S)	Class I
35/F	7	Unknown	Focal cognitive to generalized tonic	Mult	Normal	None	Noninformative	No	28	Class IV
36/F	4	Structural: FCD	Focal tonic, dystonic or clonic	F/R	F‐T‐P/R	F/R	Localizing	No	27	Class IV
37/M	13	Unknown	Two types: 1) right tonic impaired awareness, 2) left dystonic‐clonic	T/L	Normal	T‐P‐F/L	Lateralizing	No	26	Class IV
38/F	5	Structural: MTS	Two types: 1) generalized tonic, 2) myoclonic	Mult	T/R	None	Noninformative	No	25	Class IV
39/M	2	Structural: FCD	Focal tonic to GTC	F‐T/R	T/R	T/R	Highly localizing	Yes	23 (15 post‐S)	Class I
40/F	6	Structural: FCD	Sensory aura + focal clonic aware.	F/L	F/L	F/L	Highly localizing	No	22	Class II
41/F	2	Genetic: TSC	Epileptic spasms + focal clonic impaired awareness	Mult	Bilat F‐P‐T	F‐P/R	Noninformative	No	21	Class IV
42/M	12	Structural: FCD	Sensory aura + focal clonic	F‐T/L	F/L	F/R	Noninformative	No	15	Class I
43/F	15	Structural: MCA stroke + MTS	Sensory aura + focal tonic impaired awareness	F‐T/L	F‐P‐T/L	P‐O/L	Lateralizing	Yes	14 (12 post‐S)	Class I
44/M	14	Structural: FCD	Sensory aura + focal dystonic impaired awareness to GTC	P/R	P/R	P/R	Highly localizing	No	13	Class I

Abbreviations: AE, autoimmune encephalitis; AED, antiepileptic drug; ASD, autistic spectrum disorder; Bilat, bilateral; CBZ, carbamazepine; CLB, clobazam; E, epilepsy; FCD, focal cortical dysplasia; FIRES, febrile infection‐related epilepsy syndrome; GEFS+, generalized epilepsy with febrile seizures plus; GTC, generalized tonic‐clonic; HHE, hemiconvulsion‐hemiplegia encephalopathy; HSV, herpes simplex virus; iEEG, intracranial EEG; LAM, lamotrigine; LEV, levetiracetam; LGS, Lennox‐Gastaut syndrome; MCA, middle cerebral artery; MTS, mesial temporal lobe sclerosis; Mult, multifocal; OXC, oxcarbazepine; Post‐S, postsurgery; QoL, quality of life; SE, status epilepticus; SLT, sulthiame; TPM, topiramate; TSC, tuberous sclerosis complex; VNS, vagus nerve stimulator; VPA, valproic acid.

a Imaging localizations are classified as temporal (T), frontal (F), parietal (P), occipital (O) lobes, respectively, left (/L), or right (/R).

b Classification follows the current International League Against Epilepsy (ILAE) seizure and epilepsy classification.^36^

### 3.2 Feasibility study

The mean hospital stay for ictal SPECT was four days. Chronic antiepileptic medication was down‐titrated in 17/51 (33.3%) and stopped in 14/51 (27.5%) hospitalizations. Rescue medication (Lorazepam) for seizure control was needed in 12 hospitalizations (23.5%), including seven cases in which chronic AEDs were diminished or stopped and three cases that were already hospitalized for poor seizure control. Procedural sedation for ictal SPECT was adopted in 25/58 cases (43.1%). Mean age of patients receiving sedation was 4.9 years (1‐10 years), and only five patients older than 8 years received sedation. No sedation‐related complications were reported.

Fifty patients (86.2%) had their habitual seizures during injection for ictal SPECT, while eight (13.8%) had atypical seizures. Chronic antiepileptic medication management did not affect the occurrence of atypical seizures, as 31/50 (62.0%) of those with typical seizures but only 3/8 (37.5%) of those with atypical seizures had their chronic medication diminished or stopped. In 10/58 (17.2%) cases, the tracer for ictal SPECT was injected during or after a generalization of the seizure. All but one were habitual seizures. Chronic medication was modified in 7/10 of the latter.

Timings of ictal SPECT injection and acquisition are detailed in Table 2. Overall, in 53/58 (91.4%) ictal SPECT tracer injection was performed ≤30 seconds from seizure onset (first EEG/clinical ictal sign). Mean time from tracer injection to ictal SPECT acquisition averaged 40 minutes (range 13‐89 minutes). Patients requiring sedation had higher mean latency times (51 minutes) than those not requiring sedation (31 minutes).

**Table 2:** Ictal SPECT operational timings

Ictal SPECT timings	Mean	Range
From first clinical ictal sign to tracer injection	17 s	1‐65 s
From first EEG changes to injection^a^	25 s	0‐289 s
From start of injection to complete injection of the tracer	13 s	4‐46 s
From the end of injection to seizure cessation^b^	61 s	0‐390 s
From tracer injection to ictal SPECT	40 min	13‐89 min

a Injection was performed before the first EEG changes in three cases, while in one case there was no definite ictal EEG activity.

b In seven cases, seizures ended before the end of injection (mean 9 s; 3‐20 s).

Eleven patients (25.0%) were ≤6 years old (median: 3.8 years). Operational timings and feasibility variables for ictal SPECT and SISCOM in this selected age‐group are reported in Table S2. Overall, tracer injection was performed within 30 seconds in 9/12 (75.0%).

We managed to perform two consecutive ictal SPECTs during the same hospitalization in seven patients (13.7% of hospitalizations). Of these, 6/7 (85.7%) were concordant, while in one case we could get a better localization of the epileptic focus (first ictal SPECT lateralizing and second ictal SPECT localizing).

### 3.3 Efficacy study

**Figure 3:**
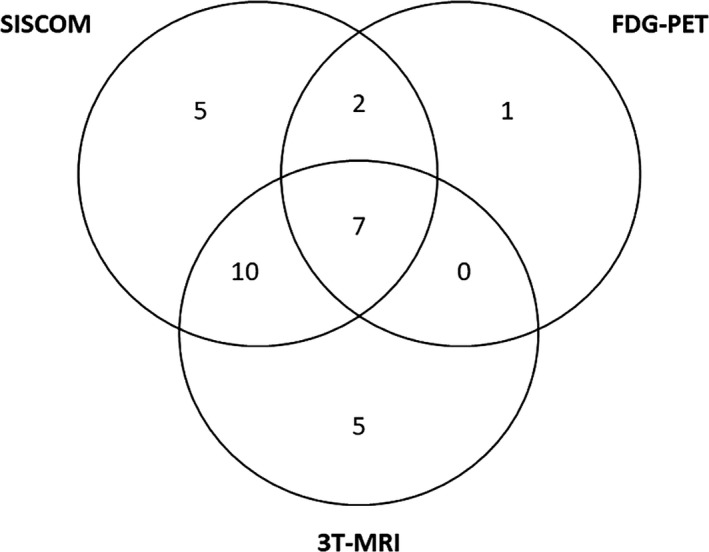
Concordance between positive outcomes of different imaging techniques. Only patients with at least a localizing or highly localizing study (positive outcome) in any imaging technique are shown (n = 44). The figure highlights the concordance in PEZ localization between the three imaging techniques (SISCOM, 3T‐MRI, and FDG‐PET) for each patient. Notice that only seven patients had a positive outcome for all three imaging techniques. Highest concordance was reached between SISCOM and 3T‐MRI (17 patients)

Imaging outcomes of the different techniques (SISCOM, MRI, ictal SPECT, and FDG‐PET) are summarized in Table 3. When comparing different techniques, SISCOM was highly localizing and significantly more localizing than ictal SPECT alone (*P* = .002), 3T‐MRI (*P* = .007), and FDG‐PET (*P* = .002). Figure 3 shows concordance in localizing and highly localizing studies between different techniques. SISCOM alone achieved better outcomes even when compared to paired techniques (considering a positive outcome when both techniques were concordant with the localization of PEZ): EEG + FDG‐PET (*P* < .001), EEG + MRI (*P* = .001), EEG + ictal SPECT (*P* = .005), MRI + ictal SPECT (*P* = .002), FDG‐PET + ictal SPECT (*P* = .021), and FDG‐PET + MRI (*P* < .001).

**Table 3:** Outcome of different imaging techniques in terms of localization of the presumed epileptogenic zone (PEZ)

Neuroimaging	Positive outcome (%)		Negative outcome (%)					
	Localizing	Highly localizing	Normal	Lateralizing	Nonconcordant	Nonlateralizing	Falsely lateralizing	Falsely localizing
SISCOM (n = 51)	11 (21.6%)	15 (29.4%)	2 (3.9%)	9 (17.6%)	6 (11.8%)	6 (11.8%)	1 (2%)	1 (2%)
MRI (n = 44)	6 (13.6%)	16 (36.4%)	13 (29.5%)	4 (9.0%)	3 (6.8%)	2 (4.5%)	0	0
ictal SPECT (n = 58)	11 (19.0%)	5 (8.6%)	3 (5.2%)	17 (29.3%)	12 (20.7%)	4 (7.0%)	2 (3.4%)	4 (7.0%)
PET (n = 20)	8 (40.0%)	2 (10.0%)	2 (10.0%)	4 (20.0%)	2 (10.0%)	2 (10.0%)	0	0

From the sixteen patients in whom the electroclinical PEZ localization was not restricted to a single lobe, SISCOM had a positive outcome in 4 (25.0%) and was lateralizing in four additional cases, while MRI had positive outcome in 3 (18.8%) and could lateralize in two more cases. Furthermore, when considering bilateral or multifocal epileptogenic foci on EEG, SISCOM showed localization (n = 2) or lateralization (n = 2) in 4/10 cases, while MRI was only lateralizing in 1/10 cases. In the eighteen patients with noninformative MRI, SISCOM had positive outcome in five cases (27.8%) and was additionally lateralizing in 5/18 (27.8%), while FDG‐PET was localizing in only three cases (16.7%).

We further analyzed our data considering SISCOM outcome as a function of potentially affecting variables (univariate analysis) including age, occurrence of habitual or atypical seizures during tracer injection, seizure generalization during injection, time from epilepsy onset, time from first ictal EEG/clinical changes and injection, and time from end of injection and seizure cessation. Among these, only the occurrence of habitual vs atypical seizures resulted in statistical significance for a positive SISCOM outcome (58.1% vs 12.5%; *P* = .018). There was no significant correlation between injection time and SISCOM outcome in our cohort. Additionally, patients with temporal lobe epilepsy had better SISCOM outcomes than patients with extratemporal epilepsies (76.9% vs 42.1% positive outcomes; *P* = .03).

Sixteen patients (16/44, 36.4%) were eventually selected for resective surgery: 12 with temporal lobe and four with frontal lobe localizations. Nine cases (56.3%) had focal cortical dysplasia, five (31.3%) had hippocampal sclerosis, and two (12.5%) had low‐grade tumors (see Table 1). Of those undergoing surgery, both SISCOM and MRI were localizing/highly localizing in 13/16 cases each (81.3%). One patient with a lateralizing SISCOM underwent functional hemispherotomy. Only two patients with a negative SISCOM outcome (nonconcordant) were selected for surgery, because of a clear localization of the PEZ on EEG and MRI (one frontal FCD and one temporal oligodendroglioma). Follow‐up of ≥12 months was available in all operated patients (median: 46 months, 8‐101 months). Of the 16 patients, 14 (87.5%) patients were seizure‐free at 12 months and thereafter. One patient achieved remission at 6 months but had relapse recurrence 10 months after surgery, after a minor head trauma (patient #11). One last patient did not achieve remission and underwent a second SISCOM one year later, documenting incomplete resection of the FCD in the right middle frontal gyrus; however, the parents refused a second surgery because of satisfactory seizure control with multiple AEDs.

Twenty‐eight patients were finally *not* considered for resective surgery. Of these, eleven did not receive surgery in spite of a positive SISCOM outcome. The reasons not to operate were the following: not eligible due to localization of the PEZ in eloquent cortex areas (n = 3), spontaneous seizure freedom achieved with one or more AEDs (n = 3), nonconcordance between SISCOM, PEZ, and other imaging techniques (n = 3), parental refusal to accept the surgical risk (n = 1, with spontaneous reduction in seizure burden), and candidate for invasive monitoring in a different specialized center (n = 1). Follow‐up was available in 27/28 of the nonoperated patients (median: 54.5 months; 13‐108 months) with the following outcome: seizure freedom was obtained in 8/27 (29.6%), mainly by the use of multiple antiepileptic drugs, seizure control (more than 50% seizure reduction from baseline) in 4/27 (14.8%), while 7/27 (25.9%) experienced poor epilepsy control (less than 50% seizure reduction reported from baseline) and 8/27 (29.6%) no seizure control, or evolution to epileptic encephalopathy. Among these patients, five were selected for vagal nerve stimulation and one for ketogenic diet.

Among the patient with a positive SISCOM outcome, 16/24 (66.7%) achieved seizure freedom and 2/24 (8.3%) seizure control (Engel I‐II 18/24 [75%]). Conversely, when SISCOM outcome was negative, only 6/19 (31.6%) had seizure remission and 4/19 (21.0%) seizure control (Engel I‐II 10/19 [52.6%]).

## 4 DISCUSSION

Pediatric data on SISCOM are scarce, and there are no studies that have systematically evaluated its feasibility in a real‐life clinical setting. In a recent meta‐analysis^17^ investigating the presurgical role of SISCOM in epilepsy, only three out of 11 selected studies were purely pediatric (81 children in total, of which 13 with TCS).^13^, ^21^, ^22^

We proved that SISCOM is feasible, even in young children. Our overall successful hospitalization rate was 71.2%, similar to other rates reported in the literature.^22^, ^23^ The main reasons for failure to perform ictal SPECT were a lack of recorded seizures and impossibility to proceed to SPECT due to seizure occurrence outside the time window in which SPECT can be performed in a clinical setting (eg, during night‐time). The two major quality outcomes for feasibility were obtained: We managed (a) to inject the radiotracer within 30 seconds from seizure onset in 91.4% of cases and (b) to perform ictal SPECT within two hours from injection in all patients. Although 43.1% of our patients received procedural sedation, this did not significantly affect feasibility (operational timings) nor SISCOM outcome. Although the need of sedation in children may rise ethical issues (as it is not harmless), we did not report complications in our cohort.

Injection delay is crucial for ictal SPECT outcome, and different studies have suggested better localizations when the radiotracer injection occurs within 30 seconds from seizure onset.^19^, ^24^ Noteworthy, injection time did not influence SISCOM outcome in this study, probably because it was performed within 30 seconds in nearly all patients. Similar results have been recently reported in a highly specialized pediatric center.^25^

Interestingly, the occurrence of atypical seizures had a significant negative impact on SISCOM outcome in our study. Epilepsy is not a static condition, especially in children, and the clinician can be wrongly oriented by caregivers when trying to define habitual seizure semiology. Therefore, it is not uncommon to record a seizure that does not completely overlap with those reported by the family. Atypical seizures may represent “aborted” seizures or be the expression of a less localized epileptogenic focus with multiple propagation pathways. Notably, AED downscaling did not correlate with an increase in atypical seizures. We therefore suggest to perform an additional ictal SPECT if tracer injection occurs during an atypical seizure based on a critical revision of the EEG.

SISCOM was able to localize or even highly localize the PEZ in 51% of cases (26/51) and to lateralize the PEZ in an additional 17.6% (9/51), achieving better outcomes than ictal SPECT, MRI, and FDG‐PET. In a similar retrospective study in 54 children with drug‐resistant epilepsy by Perissinotti et al,^22^ SISCOM was localizing in 67% of cases, showing higher concordance with the electroclinical PEZ compared to MRI (39%) and ictal SPECT (50%). The use of SISCOM improves sensitivity by avoiding possible errors related to the visual analysis of ictal SPECT alone. In light of our results, which are consistent with a large body of evidence from different studies,^13^, ^14^, ^23^, ^26^, ^27^ we recommend the use of SISCOM instead of ictal SPECT alone in the evaluation of pediatric patients with epilepsy. It should be stressed that FDG‐PET does not visualize the seizure onset zone, but the functional deficit zone. Therefore, it is used as a complementary rather than alternative technique to SISCOM.^28^, ^29^, ^30^, ^31^

In our study, SISCOM could localize the PEZ in 27.8% of the patients with noninformative MRI. In the study by Perissinotti et al, SISCOM was able to localize the PEZ in 57.6% of the patients with noninformative MRI. It is important to note that in the study by Perissinotti, MRI had a lower resolution (1.5 T) and a higher rate of noninformative studies (61%) compared to our study. In the report by Seo et al on 14 patients with noninformative MRI undergoing surgery, SISCOM showed the highest localizing value (79%) if compared with FDG‐PET and magnetoencephalography (21% and 64%, respectively), taking intracranial EEG (iEEG) as a reference.^13^ In another study using 3T‐MRI but focusing on surgically treated pediatric patients, SISCOM and MRI seemed equally precise in the localization of the epileptogenic lesion (92.3% vs 84.2%).^23^ The limitation of many retrospective studies focusing on operated patients is selection bias (patients with high multimodality concordance are more likely selected for surgery) and do not represent a truthful population of pediatric patients clinically screened for epilepsy surgery. Taken together, these results suggest that SISCOM can be reliably used in children, especially when MRI is normal or noninformative, possibly because it gives better information about the functional state of the epileptogenic area, independently from subtle anatomical/architectural changes that may be difficult to detect on MRI in this age‐group. A comprehensive multimodal approach including SISCOM in MRI‐negative patients can broaden the surgical opportunities by adding presurgical information on the localization of the PEZ, eventually guiding iEEG placement, minimizing the need for invasive studies, and reducing the craniotomy size and the number of electrodes used for iEEG.^13^, ^22^

SISCOM was of substantial utility when the PEZ was difficult to localize based on EEG and clinical records. In the present study, SISCOM could localize the PEZ in 25% of these patients, while MRI was useful in 18.8%. When considering only bilateral or multifocal epileptogenic foci on EEG, SISCOM showed localization in 20% and MRI in none. However, only 11.8% (2/17) of these patients were finally selected for resective surgery. Finally, even with the contribution of SISCOM and other imaging techniques, none of the patients with both noninformative MRI and a nonlocalized epileptogenic region on EEG underwent surgery. This is in line with other studies showing that a lack of concordance between different techniques is associated with inferior surgical outcomes.^13^, ^32^

Finally, temporal lobe localization of the PEZ correlated with better SISCOM outcomes, compared to extratemporal localizations (76.9% vs 42.1%). This ability of SISCOM to better localize temporal foci has already been reported in both adult^33^ and pediatric studies.^19^, ^32^ Depending on their origin of onset among other factors, seizures may have different propagation patterns that can influence SISCOM outcome. A possible explanation is that frontal and parietal seizures may propagate faster and more easily to large areas of the brain, resulting in poorer SPECT localizations.^7^, ^18^, ^24^

Overall, 16/44 (36.4%) patients were selected for resective surgery (twelve with temporal, four with frontal lesions). This is in line with other similar studies in which final selection for surgery was completed in almost one‐third of the patients entering the epilepsy surgery screening protocol (22%‐54%).^16^, ^19^, ^22^, ^34^ Of those undergoing surgery, both SISCOM and MRI were equally localizing the PEZ in 13/16 cases (81.3%). Globally, seizure‐free rates at 12 months were 87.5% in the operated group and 29.6% in the nonoperated group. Notably, a positive SISCOM outcome was correlated with seizure freedom in 66.7% and with a good final outcome (Engel class I‐II) in 75% of our patients. It is noteworthy that a concordance of the epileptogenic focus diagnosed by SISCOM with the surgical site can predict good surgery outcome as described in previous reports.^15^, ^27^ It has already been shown that incomplete resection of cortical dysplasia is the main predictor of poor postsurgical outcome.^35^ The very high seizure‐free rate in our cohort of operated patients is very likely due to a correct presurgical definition of the EZ and to a low frequency (12.5%; 2/16) of incomplete EZ resection.

This study holds some limitations. First, PEZ was determined on the basis of EEG and clinical data, without performing iEEG. First, as we aimed to improve noninvasive localization of the epileptic focus, we determined a hypothetical PEZ solely based on seizure semiology and EEG. There is not enough evidence to consider such PEZ solid enough from a surgical point of view. This is particularly true if we consider the clinical heterogeneity of our study population. In such cases, the anatomo‐electro‐clinical correlation is generally difficult and, consequently, the presumption of the EZ is weak. The localizing value of SISCOM is thus limited in our study by the fact that we determined the PEZ in a noninvasive way and not by iEEG which is considered the gold standard.^13^, ^17^, ^32^ Nevertheless, the EZ remains a theoretical concept that can be confirmed only after achieving postsurgical seizure freedom. The localizing value of SISCOM is indirectly supported by the fact that all operated patients had histopathologic findings consistent with epileptogenic lesions and by the high seizure freedom rate achieved after surgery in our cohort. Eventually, such results could suggest that invasive monitoring may be omitted when multiple noninvasive techniques such as MRI and SISCOM are concordant in defining the PEZ.

Second, this is a retrospective study and therefore has intrinsic limitations. Recall biases were minimized by a systematic approach to clinical records using electronic clinical data, and data analysis was performed in a blinded fashion.

## CONFLICT OF INTERESTS

None of the authors has any conflict of interest to disclose. The authors confirm that they have read the Journal's position on issues involved in ethical publication and affirm that this report is consistent with those guidelines.
