# Supplementary material for: Subtraction Ictal SPECT coregistered to MRI (SISCOM) as a guide in localizing childhood epilepsy
Source: Epilepsia Open. 2019 Dec 26;5(1):61–72. doi: 10.1002/epi4.12373 (PMC7049808; doi:10.1002/epi4.12373)
Supplement: Supplementary file 1 [file EPI4-5-61-s001.docx]

**Supplementary Data_SISCOM protocol**

All patients entering the epilepsy surgery protocol were scheduled for a 5-days hospitalization in the EEG-monitoring Unit for continuous video-EEG recording, with the aim to detect a seizure suitable for ictal-SPECT. Provocative techniques included hyperventilation, intermittent photic stimulation, sleep deprivation and down-titration or suspension of one or more AEDs.

There was no standardized universal medication management protocol. The decision either to halve or to stop one or more AEDs was based on personal epileptic and medical history: the aim is to unlock an habitual focal seizure while preventing a primarily or secondarily generalization. This can be done either before (starting fast down-titrating 2-3 days before hospitalization) or after admission. A intravenous catheter was placed in all patients at admission, connected to a shielded radionuclide tracer manual pump. Upon seizure occurrence, a trained EEG nurse would inject the radionuclide tracer ^99m^Tc-ethyl cysteinate dimer (^99m^Tc-ECD). A seizure can be detected either clinically or electroencephalographically. The patient (or the care-giver) can use a specific button, or shout “Seizure!” to signal a seizure occurrence. After tracer injection, the EEG-nurse will apply the standardized hospital medical protocol for seizures (eventually, asking for medical advice and recurring to Lorazepam 0.1mg/kg IV or Midazolam 0.1mg/kg IV in case of a primarily or secondarily generalized tonic-clonic seizure lasting more than 5 minutes). After clinical stabilization the patient was transferred to the Department of Nuclear Medicine for ictal-perfusion SPECT. Optimal timing for ictal-SPECT is between 30 and 120 minutes after injection. Tracer injection was only performed within a specific time-window of SPECT availability (i.e. Monday to Friday, from 8.30 a.m. to 4.30 p.m.). For this reason, seizures occurring outside this period are recorded but do not receive injection for ictal-SPECT. Deep sedation (Chloral hydrate, or Chlorpromazine + Pentobarbital + Promethazine) was provided for SPECT in children <8 years old and/or unable to cooperate.

Optimal injection occurs within 30 seconds from the seizure onset. Ictal-SPECT requires collaboration from SPECT images are acquired using IRIX Prism (Philips Medical Systems, Cleveland, USA) until 2010 and using Discovery NM-CT 670 (GE Healthcare) afterwards. Reconstruction of the images was performed using ordered-subset expectation maximization (OSEM) iterative reconstruction with six iterations, eight subsets without post-smoothing on IRIX-data and five iterations, ten subsets with a 3D-Gaussian post-smoothing kernel (full width at half maximum (FWHM) 6 mm) on Discovery-data. Image acquisition, reconstruction and analysis followed standardized procedures.

After ictal-SPECT, anti-epileptic drugs were up-titrated to the original dosage and the patient could be discharged if clinically stable. However, hospitalization could be extended for an additional ictal-SPECT (at least 48 hours apart, to allow a complete washout of the tracer), if necessary.

For the interictal study, radionuclide injection occurred during EEG monitoring. A seizure-free EEG activity ≥5 minutes before and ≥3 minutes after injection was required. The interictal scan was performed 20 minutes after injection, with the same procedure as for ictal-SPECT.

Ictal and interictal-SPECT studies were finally co-registered using an automatic registration algorithm based on mutual information using the registration module of Statistical Parametric Mapping (SPM version 8; Wellcome Trust Centre for Neuroimaging, UK), implemented in Matlab (R2012; The MathWorks Inc., USA). The ictal and spatially co-registered interictal images were normalized for global brain counts within each scan. The transformed, normalized interictal images were subtracted from the normalized ictal image to create an image where the value for each pixel represents the intensity difference between the two data sets. The difference image was smoothed using a 3D-Gaussian smoothing kernel (FWHM=15 mm) and transformed into a z*-*score image using the mean and standard deviation (SD) of the differences in all brain voxels. A *z*-score threshold of 2.0 was considered to have the optimal specificity and sensitivity for ictal onset zone localization^20^. The average of both SPECT images was co-registered to the structural MRI, creating a SISCOM image. The thresholded z-score map was then co-visualized onto the co-registered preoperative MRI using MRICRO software (Georgia Institute of Technology, Atlanta, USA) for detailed anatomical localization (Figure 1).

In all patients, resective surgery feasibility, strategy and eventually extent of resection were determined by multidisciplinary consensus with integration of all clinical, paraclinical and technical investigations. In some cases, ^18^FDG-PET was performed to support the hypothesis. All patients who were eligible for resective surgery in eloquent cortex areas, and able to cooperate, underwent language and/or motor functional-MRI. Informed written consent was obtained from all patients and/or their parents or legal tutors, and all procedures were approved by the Hospital Ethics Committee.
