# Supplementary material for: Subtraction Ictal SPECT coregistered to MRI (SISCOM) as a guide in localizing childhood epilepsy
Source: Epilepsia Open. 2019 Dec 26;5(1):61–72. doi: 10.1002/epi4.12373 (PMC7049808; doi:10.1002/epi4.12373)
Supplement: Supplementary file 2 [file EPI4-5-61-s002.docx]

**Table 1. Clinical and demographic characteristics**

| Patient n. | Sex | Age | Etiological background | Age at E onset | Seizures classification* | Time from E onset (months) | PEZ ^†^ | MRI ^†^ | SISCOM ^†^ | SISCOM outcome | Surgery | Pathology | Surgical eligibility/ Other therapies | Follow up (months) | Clinical outcome |
| --- | --- | --- | --- | --- | --- | --- | --- | --- | --- | --- | --- | --- | --- | --- | --- |
| 1 | F | 14 | Structural:  MTS | 10 | Focal autonomic impaired awareness | 58 | T/L | T/L | T/L | Highly-localizing | Left amygdalohippocampectomy | Hippocampal sclerosis |  | 119 (101 after-surgery) | Seizure free after surgery without AED. Mild memory impairment |
| 2 | M | 15 | Genetic:  Fragile X + refractory temporal lobe E | 7 | Focal tonic right | 103 | T/L | T/L | T/L | Highly localizing | Amygdalohippocampectomy + temporal pole | Hippocampal sclerosis |  | 117 (43 after surgery) | Seizure free after surgery without AED |
| 3 | F | 10 | Structural: FCD | 9 | Focal tonic left, impaired awareness | 12 | T/R | T/R | T/R | Localizing |  |  | Candidate for invasive monitoring | 108 | Seizure free with multiple AEDs |
| 4 | F | 9 | Structural: FCD | 8 | Focal clonic left | 5 | T/R | T/R (+ left hippocampal malrotation) | T/R | Highly-localizing | Right temporal lobectomy. Right hemispherotomy 7 months later for refractory focal SE | FCD without balloon cells |  | 106 (96 after surgery) | Seizure free after hemispherotomy |
| 5 | F | 3 | Structural: FCD + West syndrome, epilepsy relapse after corticalectomy | 0,3 | Focal tonic left | 36 | F/R | F/R | F/R | Localizing | Resection right frontal FCD (gyrus frontalis medius) | FCD without balloon cells |  | 105 | No seizure control after surgery. Parents refuse 2nd intervention. Seizure reduction on multidrug (<50%) |
| 6 | M | 12 | Structural: MTS + Auditive reflex seizures | 1 | Focal impaired awareness, with auditory aura | 132 | T/R | T/R (MTS) + P/R (suspect of FDC) | Antero-mesial T/R | Highly-localizing | Right partial anterior neocortical temporal lobectomy + amygdala and hippocampus | Hippocampal sclerosis |  | 105 (100 after surgery) | Seizure free after surgery without AED |
| 7 | M | 11 | Structural: peripartal asphyxia with left MCA stroke and brainstem hypoplasia | 0,1 | Focal impaired awareness | 135 | None | T/L (MTS) + left hemisphere hypotrophy and gliosis | T/R | Localizing |  |  | VNS | 97 | No seizure control (daily seizures) |
| 8 | M | 14 | Structural: FCD | 7 | Focal clonic aware, with aura, to bilateral tonic-clonic | 84 | F/R | F/R | F/R | Highly-localizing |  |  | Primary motor cortex involvement on iEEG (not good candidate) | 96 | Not seizure free (daily seizures) |
| 9 | M | 4 | Structural: FCD, with evolution to West syndrome | 0,3 | Focal tonic left | 52 | F/R | F/R | F/R | Highly-localizing | Resection of prefrontal right FCD | FCD, no balloon cells |  | 95 (90 after surgery) | Seizure free after surgery without AED |
| 10 | M | 7 | Immune: HHE with right hemiparesis | 0,7 | Multifocal: focal tonic, motor arrest, myoclonic | 85 | Multifocal | Left hemispheric atrophy | None | Non-lateralizing |  |  | VNS | 86 | Not seizure free (>50%) |
| 11 | M | 15 | Structural: Temporal FCD + West syndrome. Progression to focal tonic seizures | 0,6 | Focal tonic right to bilateral tonic-clonic | 172 | T/L | T/L | T/L | Localizing | Left anterior temporal lobectomy | FCD, incomplete resection |  | 79 (74 after surgery) | Ten months seizure freedom, then relapse after mild head trauma, same phenomenology |
| 12 | F | 1 | Structural: FCD with left hemiparesis and focal epilepsy | 1 | Epileptic spasms + focal non motor impaired awareness | 8 | F/R | F/R | None | Non concordant | Resection right frontal FCD | FCD type IIB |  | 78 (74 after surgery) | Seizure free after surgery without AED |
| 13 | F | 5 | Genetic: TSC | 0,4 | Multifocal: focal impaired awareness tonic to bilateral tonic-clonic and myoclonic | 58 | Multifocal | Bilateral T (FCD. No tubers) | T-P/R | Lateralizing |  |  | Everolimus | 74 | Not seizure free (<50%), multidrug + VNS treatment |
| 14 | F | 13 | Unknown: Intellectual disability | 10 | Focal tonic left | 38 | Bilateral F | Normal | T/R | Falsely localizing |  |  | VNS | 73 | Not seizure free (>50%), multidrug + VNS. Satisfactory IQ and QoL |
| 15 | M | 7 | Immune: Autoimmune encephalitis with hippocampal hypotrophy and epilepsia partialis continua | 6 | Focal clonic right | 13 | F/L | T/L | F/L | Normal |  |  |  | 72 | No seizure control (multidrug, ketogenic diet, steroids) |
| 16 | F | 9 | Unknown: Drug-resistant epileptic encephalopathy | 0,7 | Focal motor right to GTC + focal cognitive with automatism. Post ictal vomit/headache, with aphasia | 104 | Bilateral F-T | Normal | Bilateral nucleus caudatus (propagation) | Non-lateralizing |  |  |  | 71 | Period of refractory seizures, then seizure free since 12months (LAM+TPM). Cognitive regression and psychiatric comorbidity |
| 17 | F | 16 | Structural:  FCD | 0,4 | Focal onset with aura (pallor, laryngeal sensation), impaired awareness + right hand automatisms | 195 | T/R | T/R | T/R | Localizing | Right anterior temporal lobectomy and amygdalo-uncusectomy (spared hippocampus) | Architectural cortical dysplasia (uncus and amygdala) |  | 70 (64 after surgery) | Seizure free after surgery without AED |
| 18 | M | 4 | Unknown: Nocturnal frontal lobe E | 3 | Nightime hypermotor + automatisms | 8 | F/R | Normal | None | Normal |  |  |  | 68 | Seizure free since 55 months (CBZ monotherapy) |
| 19 | M | 14 | Structural: FCD | 12 | Focal impaired awareness to GTC | 17 | F/L | F/L (opercular FCD) | F/L | Localizing |  |  | High risk for aphasia (broca<1cm), parental deny | 63 | Seizure free since 45 months (VPA+LEV) |
| 20 | M | 10 | Unknown: Drug-resistant frontal lobe E | 5 | Focal aware clonic right with eyes/head deviation to right and then contralateral tonic to GTC | 54 | Bilateral F | Normal | F-P/R + propagation to thalamus/R and T/R | Lateralizing |  |  |  | 60 | Not seizure free (>50%) |
| 21 | F | 8 | Unknown: Absence E + photosensitivity: evolution to temporal lobe E with epigastric aura | 5 | Focal cognitive | 40 | Multifocal | F/R (suspect millimetric cortical heterotopia) | None | Non lateralizing |  |  |  | 56 | Not seizure free (>50%) |
| 22 | M | 11 | Structural: FCD | 9 | Focal cognitive + oral automatisms | 19 | Bilateral T | P/R (FCD) | P/R | Localizing |  |  | Not candidate, low concordance between investigations | 55 | Not seizure free (>50%) |
| 23 | M | 16 | Structural:  FCD | 13 | Focal motor impaired awareness: head and eyes to left, then to GTC | 29 | F-T/R | F/R (subcortical FCD with transmantle sign) | F-O/R | Lateralizing |  |  | Further investigations needed |  |  |
| 24 | F | 8 | Immune:  FIRES + evolution to focal E | 6 | Focal motor right (+ left) clonic with impaired awareness | 22 | F/L | Normal | F-T/L | Lateralizing |  |  | Parental deny for invasive monitoring/surgery. VNS | 54 | Seizure reduction (<50%) multidrug + VNS |
| 25 | F | 6 | Structural:  FCD | 3 | Focal autonomic impaired awareness | 27 | T/R | T/R | low antero-T/R + propagation to striatum/R | Highly-localizing | Anterior right temporal lobectomy + partial amygdalohippocampectomy | Cortical dysplasia type IIB |  | 51 (41 after surgery) | Seizure free after surgery without AED |
| 26 | M | 12 | Genetic: Klinefelter syndrome + focal E with cognitive regression | 2 | Focal cognitive + left clonic | 120 | T/R | T/R | T/R + propagation basal ganglia | Localizing | Right temporal lobectomy | Ganglioglioma WHO grade II |  | 50 (46 after surgery) | Seizure free after surgery without AED |
| 27 | F | 7 | Genetic:  PAI syndrome | 4 | Focal sensitive, than tonic right | 43 | P/L | Normal | F-P/L | Lateralizing |  |  | Cadidate for invasive monitoring | 50 | Seizure reduction (<50%) with OXC+CLB |
| 28 | M | 8 | Unknown: Alice in wonderland syndrome | 7 | Left focal clonic aware (EEG onset on vertex) | 6 | F-P/R | Normal | High P/R | Highly-localizing |  |  | Cadidate for invasive monitoring | 44 | Seizure free since 39 months (SLT monotherapy) |
| 29 | M | 12 | Unknown: Nocturnal E | 7 | Right tonic to GTC | 60 | F/L | Normal | Mid-F on the midline | Highly-localizing |  |  | Candidate invasive monitoring | 43 | Seizure free since 13 months (monotherapy VPA). |
| 30 | F | 7 | Unknown: Focal E | 3 | Focal clonic with left Todd's paresis | 54 | F/R | Normal | high -F/R + right insular-mesiotemporal (propagation) | Highly-localizing |  |  | Suspected primary motor facial, but sensory aura not excluded | 39 | Not seizure free (>50%) |
| 31 | M | 13 | Unknown: Focal E | 4 | Focal non motor impaired awareness | 109 | F-T/R | Normal | F-P/L on the midline | Non concordant |  |  |  | 38 | Seizure reduction (<50%) multidrug |
| 32 | M | 10 | Structural:  Hippocampal tumor | 8 | Focal sensory aware | 30 | T/R | T/R (hippocampal lesion) | Bilateral T | Non concordant | Anterior temporal lobectomy and amygdalohippocampectomy | Oligodendroglioma WHO grade II |  | 34 (33 after surgery) | Seizure free after surgery without AED. Good cognitive outcome |
| 33 | M | 10 | Structural: MTS | 2 | Focal motor impaired awareness | 113 | T/R | T/R (MTS) | Mesial T/R | Highly-localizing | Right anterior temporal lobectomy with amygdalohippocampectomy | Hippocampal sclerosis |  | 32 (25 after surgery) | Seizure free after surgery without AEDs. Cognitive improvement, working memory deficit |
| 34 | M | 15 | Structural: FCD | 0,7 | Nocturnal focal hyperkinetic | 179 | F/R | F/R (FCD with transmantle sign) | High-F/R + propagation to talamo-striatal/R | Highly-localizing | FCD resection (mesial cortex SMA-pre-SMA paracentralis, precuneus, g cinguli) | Cortical dysplasia type IIA |  | 31 (12 after surgery) | Seizure free |
| 35 | F | 7 | Unknown: Myoclonic E at 2 years + evolution to LGS | 2 | Focal cognitive and generalized tonic seizures | 60 | Multifocal | Normal | Right hemisphere hyperperfusion | Non lateralizing |  |  |  | 28 | No seizure control |
| 36 | F | 4 | Structural: FCD | 3 | Left focal tonic, dystonic or clonic | 17 | F/R | F-T-P/R (large FCD with abnormal gyration) | F/R + propagation to P-T/R and basal ganglia/R | Localizing |  |  | Indication to invasive monitoring | 27 | No seizure control |
| 37 | M | 13 | Unknown: GEFS+ | 12 | Two types: 1) right tonic impaired awareness + head version; 2) left dystonic-clonic | 19 | T/L | Normal | T-P-F/L | Lateralizing |  |  | VNS | 26 | No seizure control |
| 38 | F | 5 | Structural: HSV encephalitis with right MTS | 3 | Two types: 1) generalized tonic; 2) head myoclonic | 11 | Multifocal | T/R (MTS) | None | Non lateralizing |  |  |  | 25 | No seizure control, evolution to epileptic encephalopathy |
| 39 | M | 2 | Structural:  FCD | 1 | Right focal tonic to GTC + postictal left facial palsy | 12 | F-T/R | T/R | Anterior T/R | Highly-localizing | Resection right temporal FCD | FCD type IIA |  | 23 (15 after surgery) | Seizure free after surgery, without AED |
| 40 | F | 6 | Structural: FCD | 3 | Focal sensitive aura then right clonic aware. Postictal hemiparesis | 48 | F/L | F/L (FCD gyrus precentralis) | High F/L (gyrus precentralis on the midline) | Highly-localizing |  |  | Parental refusal | 22 | Seizure contol (<50%), OXC+TPM |
| 41 | F | 2 | Genetic: TSC + West syndrome | 0,5 | Epileptic spasms + focal right clonic impaired awareness | 19 | Multifocal | Multifocal (bilateral fronto-parietal and temporal subcortical tubers) | F-P/R | Falsely lateralizing |  |  | Ketogenic diet | 21 | No seizure control, encephalopatic evolution |
| 42 | M | 12 | Structural: FCD | 12 | Right focal clonic with sensitive aura, aware + postictal aphasia and Todd's paresis | 2 | F-T/L | F/L (FCD type II from lateral ventricle to opercular cortex) | F/R (orbitofrontal) + propagation to latero-T/R | Non-concordant |  |  | Hiih risk for postop aphasia, surgery is eventually postponed to adulthood | 15 | Seizure free on AED (multidrug) |
| 43 | F | 15 | Structural: left MCA stroke | 13 | Focal impaired awareness tonic right + sensory aura (stomach-ache) | 32 | F-T/L | left MCA infarction with poroencephalic cyst and degeneration | P-O/L (posterior to the cystic formation) | Lateralizing | Functional hemisferotomy (callosotomy+temporal lobectomy+frontobasal disconnection) | Hippocampal sclerosis |  | 14 (12 after surgery) | Seizure free after surgery, without AEDs |
| 44 | M | 14 | Structural: FCD | 12 | Sensitive aura left arm + focal dystonic impaired awareness to GTC | 24 | P/R | P/R (FCD type II postcentral gyrus) | Lateral high-P/R | Highly-localizing |  |  | High risk of postop propioceptive deficits left arm/hand | 13 | Seizure free on AED |

^†^ Imaging localizations are classified as temporal (T), frontal (F), parietal (P), occipital (O) lobes, respectively left (/L) or right (/R).

*Classification follows the current International League Against Epilepsy (ILAE) seizure and epilepsy classification (RS. Fisher et al. Epilepsia 2017).

AED: Antiepileptic drug; ASD: Autistic spectrum Disorder; CBZ: Carbamazepine; CLB: Clobazam; E: Epilepsy; FCD: Focal cortical dysplasia; FIRES: Febrile infection-related epilepsy syndrome; GEFS+: Generalised epilepsy with febrile seizures plus; GTC: Generalized tonic-clonic; HHE: Hemiconvulsion-Hemiplegia encephalopathy; HSV: Herpes Simplex virus; iEEG: intracranial EEG; LAM: Lamotrigine; LEV: Levetiracetam; LGS: Lennox-Gastaut Syndrome; MCA: Middle Cerebral Artery; MTS: Mesial temporal lobe sclerosis; OXC: Oxcarbazepine; QoL: Quality of life; SE: status epilepticus; SLT: Sulthiame; TPM: Topiramate; TSC: Tuberous Sclerosis Complex; VNS: Vagus Nerve Stimulator; VPA: Valproic acid.
