# Supplementary material for: Subtraction Ictal SPECT coregistered to MRI (SISCOM) as a guide in localizing childhood epilepsy
Source: Epilepsia Open. 2019 Dec 26;5(1):61–72. doi: 10.1002/epi4.12373 (PMC7049808; doi:10.1002/epi4.12373)
Supplement: Supplementary file 3 [file EPI4-5-61-s003.docx]

**Table 5_supplementary data. Feasibility of SISCOM in children ≤6 years old.**

Operational timings and feasibility variables in a total of 12 SISCOM performed in 11 children younger than six years.

| Age (years),  median [range] | Delay from epilepsy onset to SISCOM (months),  median [range] | Injection during habitual seizure,  n./total (%) | Injection during generalization,  n./total (%) | Chronic AED diminished or stopped, n./total (%) | Procedural sedation, n./total (%) | Rescue antiepileptic treatment,  n./total (%) | Time from first EEG changes to injection (seconds), median [range] | Time from first clinical changes to injection (seconds), median [range] | Localizing SISCOM,  n./total (%) | Lesional MRI,  n./total (%) | Selected for surgery,  n./total (%) |
| --- | --- | --- | --- | --- | --- | --- | --- | --- | --- | --- | --- |
| 3.82 [1-6] | 22.5 [8-58] | 9/12 (75%) | 3/12 (25%) | 7/12 (58%) | 11/12 (92%) | 1/12 (8%) | 15 [3-289] | 12 [3-50] | 7/12 (58%) | 10/11 (91%) | 5/11 (45%) |

AED: antiepileptic drugs; Injection: radiotracer injection for ictal-SPECT
